# Human-Induced Pluripotent Stem-Cell-Derived Smooth Muscle Cells Increase Angiogenesis to Treat Hindlimb Ischemia

**DOI:** 10.3390/cells10040792

**Published:** 2021-04-02

**Authors:** Xixiang Gao, Mingjie Gao, Jolanta Gorecka, John Langford, Jia Liu, Jiesi Luo, Ryosuke Taniguchi, Yutaka Matsubara, Hao Liu, Lianrui Guo, Yongquan Gu, Yibing Qyang, Alan Dardik

**Affiliations:** 1 Department of Vascular Surgery, Xuanwu Hospital, Capital Medical University and Institute of Vascular Surgery, Capital Medical University, Beijing 100053, China; xixiang.gao@xwh.ccmu.edu.cn (X.G.); lianrui.guo@hotmail.com (L.G.); guyqvip@sina.com (Y.G.); 2 Vascular Biology & Therapeutics Program, Yale School of Medicine, New Haven, CT 06519, USA; mingjie.gao@xwh.ccmu.edu.cn (M.G.); jolanta.gorecka@yale.edu (J.G.); john.t.langford@yale.edu (J.L.); jia.liu.jl3365@outlook.com (J.L.); jiesi.luo@yale.edu (J.L.); ryosuke.taniguchi@yale.edu (R.T.); matsubara.yutaka.391@m.kyushu-u.ac.jp (Y.M.); liuhao73@smu.edu.cn (H.L.); yibing.qyang@yale.edu (Y.Q.); 3 Department of Surgery, Yale School of Medicine, New Haven, CT 06519, USA; 4 Department of Vascular Ultrasound, Xuanwu Hospital, Capital Medical University, Beijing 100053, China; 5 Yale Cardiovascular Research Center, Section of Cardiovascular Medicine, Department of Internal Medicine, Yale School of Medicine, New Haven, CT 06511, USA; 6 Yale Stem Cell Center, Yale University, New Haven, CT 06520, USA; 7 Department of Pathology, Yale University, New Haven, CT 06520, USA; 8 Department of Surgery and Sciences, Kyushu University, Fukuoka 812-8582, Japan; 9 Department of Cellular and Molecular Physiology, Yale School of Medicine, New Haven, CT 06520, USA; 10 Department of Surgery, VA Connecticut Healthcare System, West Haven, CT 06516, USA

**Keywords:** induced pluripotent stem cells, smooth muscle cells, angiogenesis, peripheral artery disease, chronic limb-threatening ischemia

## Abstract

Induced pluripotent stem cells (iPSC) represent an innovative, somatic cell-derived, easily obtained and renewable stem cell source without considerable ethical issues. iPSC and their derived cells may have enhanced therapeutic and translational potential compared with other stem cells. We previously showed that human iPSC-derived smooth muscle cells (hiPSC-SMC) promote angiogenesis and wound healing. Accordingly, we hypothesized that hiPSC-SMC may be a novel treatment for human patients with chronic limb-threatening ischemia who have no standard options for therapy. We determined the angiogenic potential of hiPSC-SMC in a murine hindlimb ischemia model. hiPSC-SMC were injected intramuscularly into nude mice after creation of hindlimb ischemia. Functional outcomes and perfusion were measured using standardized scores, laser Doppler imaging, microCT, histology and immunofluorescence. Functional outcomes and blood flow were improved in hiPSC-SMC-treated mice compared with controls (Tarlov score, *p* < 0.05; Faber score, *p* < 0.05; flow, *p* = 0.054). hiPSC-SMC-treated mice showed fewer gastrocnemius fibers (*p* < 0.0001), increased fiber area (*p* < 0.0001), and enhanced capillary density (*p* < 0.01); microCT showed more arterioles (<96 μm). hiPSC-SMC treatment was associated with fewer numbers of macrophages, decreased numbers of M1-type (*p* < 0.05) and increased numbers of M2-type macrophages (*p* < 0.0001). Vascular endothelial growth factor (VEGF) expression in ischemic limbs was significantly elevated with hiPSC-SMC treatment (*p* < 0.05), and inhibition of VEGFR-2 with SU5416 was associated with fewer capillaries in hiPSC-SMC-treated limbs (*p* < 0.0001). hiPSC-SMC promote VEGF-mediated angiogenesis, leading to improved hindlimb ischemia. Stem cell therapy using iPSC-derived cells may represent a novel and potentially translatable therapy for limb-threatening ischemia.

## 1. Introduction

Peripheral artery disease (PAD) affects at least 8.5 million people in the United States [1] and more than 200 million people worldwide [2]. PAD causes a range of clinical manifestations, including intermittent claudication (IC) and chronic limb-threatening ischemia (CLTI), leading to rest pain, tissue damage, ulceration, and gangrene. It is estimated that there are 6.5 million patients with CLTI in the US, Europe, and Japan based on global population-based studies [3]. Treatment options for patients with CLTI are usually limited to revascularization, including surgical and endovascular methods. Unfortunately, current interventions are unfeasible in many patients because extensive disease precludes effective revascularization [4]. About 20% of CLTI patients (‘no-option CLTI’) are not suitable for classical treatments due to a high postoperative re-occlusion rate, poor anatomical conditions, or severe co-morbidity [5]. Therapeutic angiogenesis offers the potential to restore blood supply to ischemic tissue and prevent amputation in patients who otherwise have no options for revascularization [6]. However, trials of therapeutic angiogenesis using single factors have not been uniformly efficacious or widely adopted for clinical use [7].

Stem cell therapy is thought to be another strategy to treat patients with ‘no-option CLTI.’ In particular, adult-derived stem cells, such as mesenchymal stem cells (MSC) show efficacy in several clinical trials [8,9,10,11]. A retrospective study with a 10-year follow-up of 59 patients with critical limb ischemia due to thromboangiitis obliterans in China showed that amputation-free survival was 85.3% (29/34) with stem cell therapy compared with 40% (6/15) in patients treated with aspirin (*p* = 0.0019); ulcer area, toe-brachial index, transcutaneous oxygen pressure, and pain score were also significantly improved with stem cell therapy [12]. However, adult-derived cells require invasive harvesting of tissue such as bone marrow or adipose tissue and are incapable of repopulating many cell lineages, as well as being typically delivered in an inactive state. Induced pluripotent stem cells (iPSC) are a clinically relevant and newer cell type with potential application for therapeutic angiogenesis. Since iPSC can be reprogrammed, in principle, from any adult somatic cells, such as fibroblasts, cell collection does not require invasive biopsies, and the potential pool of source cells is much greater than other stem cell types [13,14]. iPSC can be differentiated into virtually any adult cell lineage with an essentially unlimited supply of cells of interest.

Although iPSC are exciting and promising for multiple therapies, clinical application of iPSC is limited by potential teratogenicity when undifferentiated, potential genomic disruption during reprogramming protocols, and potential for low differentiation efficiencies [13]. As such, thus far, human iPSC (hiPSC)-derived endothelial cells (hiPSC-EC) and hiPSC-derived MSC have shown attenuated hindlimb ischemia in preclinical models [15,16,17]. Vascular smooth muscle cells (SMC) are critical for vascular function since they provide contractile function and structural support to blood vessels. We previously showed that delivery of hiPSC-SMC increases angiogenesis and accelerates diabetic wound healing in a murine model [18], suggesting that hiPSC-SMC may be have potential for translation to human therapeutic angiogenesis. Although protocols to differentiate hiPSC into SMC are more efficient and yield a more uniform cellular population compared with protocols to produce hiPSC-EC, few studies thus far have examined the angiogenic potential of hiPSC-SMC in a hindlimb ischemia model [19]. To increase the translational potential of these findings, we used a mouse hindlimb ischemia model to determine whether hiPSC-SMC ameliorate ischemia. We hypothesized that hiPSC-SMC promote angiogenesis and improve hindlimb ischemia; we used human adipose-derived mesenchymal stem cells (ADSC) as a positive control group [18,20].

## 2. Materials and Methods

### 2.1. Derivation of hiPSC-SMC

Human cell populations were derived using protocols approved by the Yale University Human Investigation Committee. Briefly, previously established human-induced pluripotent stem cell (hiPSC) line Y6 was used [21,22]. With approval from the Yale University Institutional Review Board, Y6 hiPSC were produced by reprogramming fibroblasts that were derived from discarded female neonatal skin tissue using Sendai viral particles that encode human OCT4, KLF4, SOX2, and c-MYC genes (Thermo Fisher, Waltham, MA, USA). Pluripotency was maintained by expanding hiPSC in mTeSR1 medium (StemCell Technologies, Cambridge, MA, USA) on Growth Factor Reduced (GFR)-Matrigel (Corning)-coated plates using feeder-free conditions at 37 °C; cells were subcultured every 5–7 days using ethylenediaminetetraacetic acid (EDTA; Thermo Fisher, Waltham, MA, USA).

The derivation of smooth muscle cells (SMC) from hiPSC used an embryoid body (EB) formation-driven strategy that we previously described [23]. Briefly, hiPSC were expanded until they achieved 80% confluency, and then they were dissociated to form cell clusters. The cell clusters were then cultured in suspension using a 6-well low attachment plate in mTeSR1 medium for 24 h. The culture medium was transitioned gradually to EB differentiation medium (DMEM containing 10% fetal bovine serum (FBS)) in order to promote EB formation until day 6. The EB were then transferred and cultured in gelatin-coated plates for 6 days in EB differentiation medium. Differentiated cells were then harvested, seeded onto Matrigel-coated plates, and cultured using smooth muscle growth medium (SmGM-2 medium, Lonza, Basel, Switzerland) for another 7–10 days in order to derive proliferative hiPSC-SMC (hiPSC-SMC-P). To promote the maturation of hiPSC-SMC, hiPSC-SMC-P were further seeded on gelatin-coated plates and cultured in SMC maturation medium (DMEM medium containing 1% FBS and 1 ng/mL TGFβ1) for 5–7 days to obtain mature hiPSC-SMC (hiPSC-SMC-M).

### 2.2. Human Vascular Smooth Muscle Cells

Primary human vascular SMC (Lonza, Basel, Switzerland) were expanded in SmGM-2 on 0.1% (*w/v*) gelatin (Sigma-Aldrich, St. Louis, MO, USA)-coated culture dishes (37 °C). Cells were passaged at 80% confluency using 0.05% trypsin-EDTA. To induce the maturation phenotype of primary SMC, primary SMC grown in SmGM-2 medium were subcultured using SMC maturation medium for seven days.

### 2.3. Human ADSC Cell Culture

Human ADSC (EMD Serono, Billerica, MA, USA; Lot VP1708071) were obtained from an adult healthy donor. ADSC (from passage 2) were cultured (24 h) to reach ~90% confluence. ADSC passages 3–4 were maintained in ADSC basal medium, which included the ADSC-GM SQ Kit (Lonza, Basel, Switzerland). Prior to experiments, the medium was changed to FBS-free medium for 12 h.

### 2.4. Quantitative PCR

RNA was extracted from cells, and a quantitative reverse transcription PCR (qPCR) assay was performed to evaluate gene expression. RNA extraction and purification used the TRIzol RNA Isolation Kit (Thermo Fisher, Waltham, MA, USA) according to the manufacturer’s instructions. Total RNA was then treated with reverse transcription using the iScript cDNA synthesis Kit (Bio-rad, Hercules, CA, USA). Primer sequences of the genes used for qPCR are listed in Table A1. qPCR was performed using the Bio-Rad IQ SYBR green supermix. Gene expression was normalized to human GAPDH. Three biological replicates were used for each analysis.

### 2.5. Animal Model

All animal studies were performed in compliance with federal guidelines and approved by Yale University’s Institutional Animal Care and Use Committee. Male athymic nude mice (Foxn1^nu^, 8–12 weeks; 20–30 g; Jackson Laboratory, ME) were used in the hind limb ischemia model as previously described [24,25]. Mice were anesthetized with vaporized isoflurane. The right hind limb was visualized using a dissecting microscope, and a 1-cm longitudinal skin incision was made from the inguinal region toward the knee. Subcutaneous tissue superficial to the neurovascular bundle was dissected. The membranous femoral sheath was gently opened to expose the neurovascular bundle, and the femoral nerve was preserved. The common femoral artery, deep femoral artery, popliteal artery and saphenous artery were carefully dissected and ligated with 10-0 sutures, and the intervening segment between sutures was divided. The skin incision was closed using 6-0 nylon sutures, and the mouse was allowed to recover inside a recovery cage. At the time of surgery, the mice received intramuscular injections of either 1 × 10^6^ hiPSC-SMC in PBS, 1 × 10^6^ ADSC in PBS, or PBS without cells. The total volume of each treatment was 100 μL, given as two 25 μL injections at two sites (distal and proximal) in both the adductor muscle and the gastrocnemius muscle. In some mice, SU5416, a potent and selective inhibitor of the Flk-1/KDR receptor tyrosine kinase (APExBIO Technology LLC, Houston, TX, USA) in DMSO (50 μL) or DMSO alone (vehicle control) was injected intraperitoneally daily, starting from day 0.

**Figure A1:**
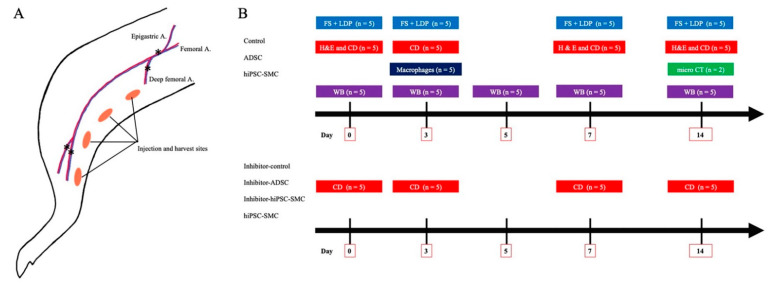
Methods. (**A**), hind limb ischemia model. The asterisks (*) denote the site of ligation, and the orange ovals denote the iPSC-SMC injection sites and muscle harvest sites. (**B**), Flow chart showing the experimental design of this study. FS: functional scoring; LDP: Laser Doppler imaging; CD: capillary density; WB: Western blotting; Inhibitor; VEGF receptor 2 tyrosine kinase inhibitor SU5416.

At the end of the study, the mice were euthanized, and the adductor and gastrocnemius muscles were harvested and submerged in 10% phosphate-buffered formalin overnight. The samples were then paraffin embedded, sectioned serially every 5 μm, and processed further as described below. The experimental design of this study is shown in Figure A1.

### 2.6. Functional Scoring

Mice were examined immediately postoperatively after recovery from anesthesia, and at 3, 7 and 14 days after induction of hind limb ischemia. Functional grading was performed according to the Tarlov scale [25,26] and the Faber hind limb ischemia score [27,28] (Table 1).

### 2.7. Laser Doppler Perfusion Imaging

Laser Doppler imaging (LDI) was performed before, immediately after, and 3, 7 and 14 days after surgery on both the ischemic and control hindlimb using an LDI2-HR Laser Doppler Imaging System (Moor Instruments; Wilmington, DE, USA) as previously described [24]. Briefly, mice were anesthetized lightly in an induction chamber containing 2% isoflurane in 100% oxygen with a flow rate of 2 L/min. Mice were then connected via a nasal cannula to a continuous flow of isoflurane/oxygen and placed on a heating pad to maintain 35–37 °C; images of both limbs were analyzed in both supine and prone positions using Moor LDI software. The region of interest was the lateral gastrocnemius muscle and the foot as this accounts for the majority of leg perfusion [25]. To account for variation in ambient light, temperature, and arterial pressure, the perfusion index was expressed as the ratio of the ischemic to the control hindlimb.

### 2.8. MicroCT Imaging

To visualize the arterial collateral network after femoral artery excision, we performed microCT imaging as previously described [29,30]. Briefly, 14 days after surgery, the abdominal aorta was cannulated and perfused with vasodilation buffer (e.g., PBS with papaverine (Paveron, Weimer, Germany) 4 mg/L, adenosine 1 g/L) for about 3 min with 100–120 mm H_2_O pressure, followed by perfusion with contrast (20% bismuth nanoparticles with 10% gelatin (Sigma-Aldrich, St. Louis, MO, USA) and PBS; 0.2 mL/10 g body weight) at 0.75 mL/min. The hindlimb vascular network morphology was imaged and analyzed using a high resolution microCT imaging system.

### 2.9. Immunofluorescence

Immunofluorescence was performed as previously described [31]. Slides were de-paraffined using xylene and then treated with a graded series of ethanol washes. Sections were heated in citric acid buffer (pH 6.0, 100 °C, 10 min) for antigen retrieval and subsequently blocked with 5% bovine serum albumin in PBS containing Tween (PBST) for 1 h (room temperature) prior to an overnight incubation (4 °C) with the primary antibodies diluted in PBST.

Sections were then incubated with secondary antibodies at room temperature (1 h). Sections were stained using SlowFade^®^ Gold Antifade Mountant with DAPI (Invitrogen, Carlsbad, CA, USA) and a coverslip applied. Antibodies are listed in Table A2.

For cell staining, cells were cultured in a 12-well plate on glass coverslips until ~50% confluence, at which time they were fixed using 4% paraformaldehyde (30 min, room temperature) and processed as described above.

Digital fluorescence images were captured, and positive cells were counted manually using ImageJ software (NIH, version 1.8.0). To determine the number of positive cells, four sections were chosen at random, cells were stained with DAPI, and signals of interest were counted and averaged. For the analysis of macrophage subtypes, the percentage of dual-positive cells to CD68 positive cells was used.

### 2.10. Capillary Density Analysis

Mice were sacrificed at 0, 1, or 2 weeks after surgery; the adductor and gastrocnemius muscles were harvested, fixed in 10% phosphate-buffered formalin, and embedded in paraffin. Five-micron sections were used. Muscle fiber number and size were examined in sections that were stained with hematoxylin and eosin; counts of five separate fields in four distinct areas were averaged for each specimen. Capillary density was assessed using immunofluorescence withher649-conjugated GSL I-B4 isolectin (Vector Laboratories, Burlingame, CA, USA). Capillary density was measured using image analysis (MetaMorph software, Molecular Devices, San Jose, CA, USA, Version 7.7.2.0) in five different randomized fields.

### 2.11. Western Blotting

Tissue lysates were extracted using RiPA Lysis buffer (Millipore, Burlington, MA, USA). Equal amounts of protein (20–40 μg) were loaded and separated using a 10% SDS-PAGE gel and electrophoretically transferred onto polyvinylidene difluoride microporous membranes (0.45 μm pore size, Millipore, Sigma-Aldrich, St. Louis, MO, USA). Membranes were blocked using TBS with Tween 20 (TBS-T) containing 5% bovine serum albumin (1 h, room temperature) and incubated with anti- vascular endothelial growth factor (VEGF)-A (1:1000; Santa Cruz, Dallas, TX, USA) or HSP-90 (1: 10,000, Santa Cruz, Dallas, TX, USA) primary antibodies overnight (4 °C) on a shaker. After the membranes were washed with TBS-T and incubated with either anti-rabbit or anti-mouse horseradish peroxidase-conjugated secondary antibody (Cell Signaling, Beverly, MA, USA) (1 h, room temperature), immunocomplexes were visualized using chemiluminescence (GE Healthcare, Chicago, IL, USA) according to the manufacturer’s instructions.

### 2.12. Statistics

Data are expressed as the mean ± standard error of the mean (SEM). Data were determined to be from a normal distribution with equal variance using the Shapiro-Wilk test. Statistical analysis was performed using either Student’s t-test to compare two groups or analysis of variance followed by Tukey’s post hoc test for multiple comparisons; one-way ANOVA was performed unless a two-way ANOVA test was specifically indicated, using GraphPad Prism version 8.0 (La Jolla, CA, USA). A *p*-value < 0.05 was considered significant.

## 3. Results

### 3.1. Characterization of hiPSC-SMC

**Figure 1:**
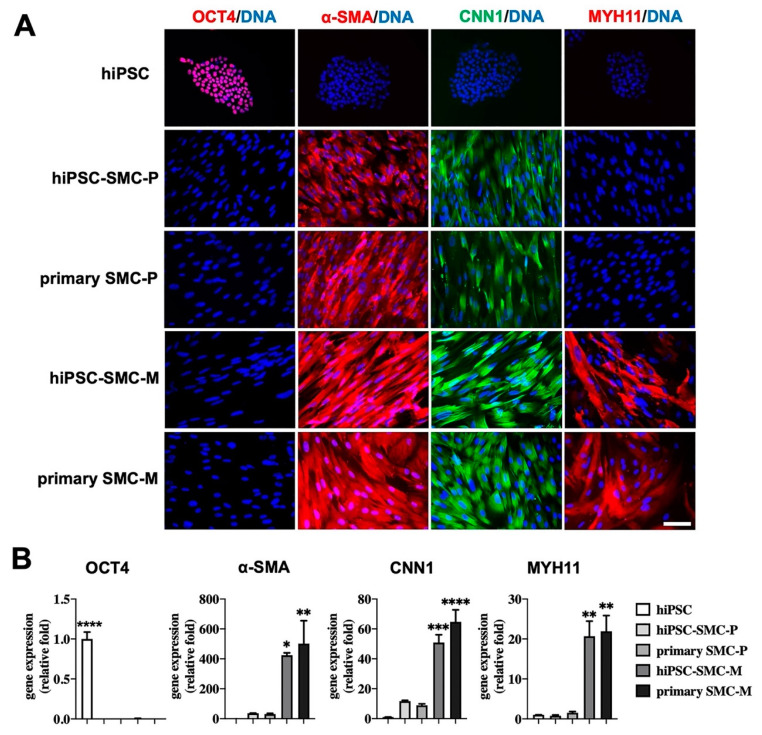
Characterization of hiPSC-SMC. (**A**) Immunofluorescence of the pluripotency marker OCT4 and the SMC-specific markers α-SMA, calponin 1 (CNN1) and smooth muscle myosin heavy chain 11 (MYH11) in hiPSC, proliferative hiPSC-SMC (hiPSC-SMC-P), proliferative primary human SMC (primary SMC-P), mature hiPSC-SMC (hiPSC-SMC-M), and mature human SMC (primary SMC-M). Scale bar, 200 μm. (**B**) Bar graphs show qPCR analysis of relative transcript numbers of OCT4 and SMC-specific marker genes in hiPSC, hiPSC-SMC-P, primary SMC-P, hiPSC-SMC-M, and primary SMC-M. Values on the *y*-axis represent fold changes relative to number of human GAPDH transcripts; gene expression is relative to that in hiPSC (ANOVA with Tukey’s multiple comparisons test); *n* = 3. * *p* < 0.05; ** *p* < 0.01; *** *p* < 0.001; **** *p* < 0.0001.

To determine the effects of iPSC-derived differentiated cells on hindlimb ischemia, we used hiPSC-SMC, a cell derivative that we have previously shown to have efficacy in wound healing [18]. hiPSC-derived SMC grown in SmGM-2 growth medium were early stage, proliferative SMC (hiPSC-SMC-P) that expressed SMC markers including α-SMA and calponin (Figure 1A,B). These early-stage hiPSC-SMC did not show expression of pluripotent markers OCT4, similar to proliferative human primary SMC (primary SMC-P; Figure 1A,B). hiPSC-VSMC-P were then matured using a low concentration of serum and TGFβ1 without EGF, bFGF or insulin; mature hiPSC-SMC (hiPSC-SMC-M) showed significantly increased expression of SMC markers including α-SMA, CNN1 and MYH11, which was comparable to the gene expression pattern of mature human primary SMC (primary SMC-M; Figure 1A,B).

### 3.2. hiPSC-SMC Ameliorate Ischemia In Vivo

**Figure 2:**
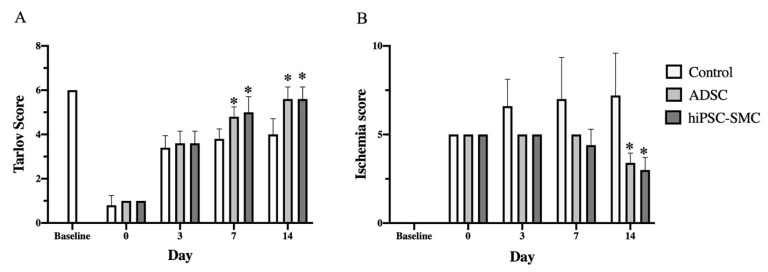
hiPSC-SMC improve postoperative functional scores. (**A**) Bar graph shows the Tarlov score in mice treated with control, ADSC or hiPSC-SMC (two-way ANOVA, *p* < 0.05; *n* = 5, * *p* < 0.05 (post-hoc)). (**B**) Bar graph shows the Faber ischemia score in mice treat with control, ADSC or hiPSC-SMC (two-way ANOVA, *p* < 0.01; *n* = 5, * *p* < 0.05 (post-hoc)).

**Figure 3:**
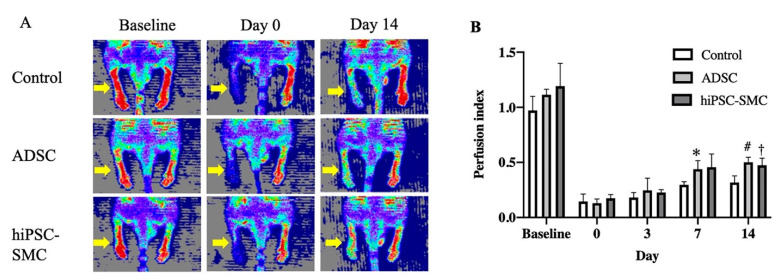
hiPSC-SMC ameliorate hind limb ischemia. (**A**) Representative laser Doppler images showing perfusion in mice treated with control, ADSC or hiPSC-SMC. (**B**) Bar graph shows the blood perfusion index of different groups; the blood perfusion index is the ratio of perfusion in the ischemic limb (right side with yellow arrows) to the nonischemic limb (left side) (two-way ANOVA, *p* = 0.001; *n* = 5, * *p* = 0.024, † *p* = 0.054, # *p* = 0.002 (post-hoc)).

**Figure A2:**
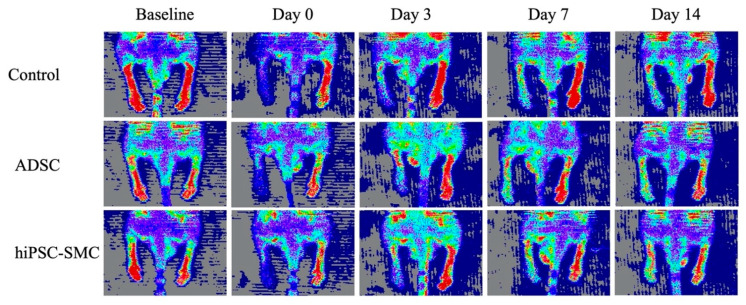
ADSC and hiPSC-SMC ameliorate hind limb ischemia. Representative laser Doppler images showing perfusion in mice treated with control, ADSC or hiPSC-SMC.

To determine whether hiPSC-SMC ameliorate ischemia, we injected hiPSC-SMC intramuscularly into the ischemic limbs of nude mice at two sites (distal and proximal) in both the adductor muscle and the gastrocnemius muscle, and PBS alone was injected as a negative control. Human ADSC were also used as a positive control since they have the potential to increase angiogenesis [32], and we have previously shown their ability to achieve wound healing in nude mice [18]. Both hiPSC-SMC- and ADSC-treated mice showed improved functional outcomes compared with control-treated mice, with increased Tarlov scores at day 7 and day 14 (*p* < 0.05, Figure 2A), as well as decreased Faber ischemia scores at day 14 (*p* < 0.05, Figure 2B). Laser Doppler images showed improved perfusion in hiPSC-SMC- and ADSC-treated mice compared with control mice at day 14 (hiPSC, 47.4 ± 3.7%, *p* = 0.054; ADSC, 50.2. ± 2.0%, *p* = 0.002; Figure 3 and Figure A2).

**Figure 4:**
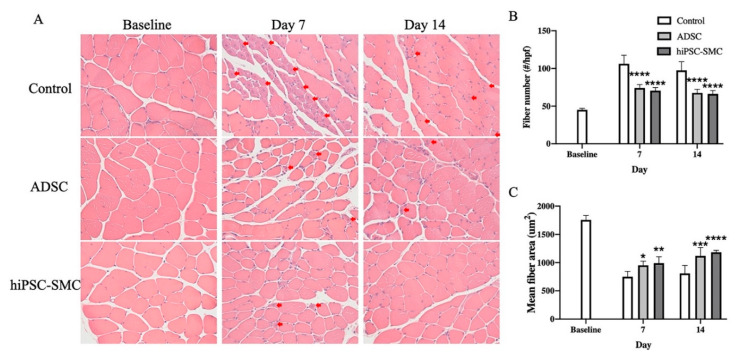
hiPSC-SMC improve gastrocnemius muscle histology. (**A**) Representative photomicrographs showing histology gastrocnemius muscle of mice treated with PBS, ADSC or hiPSC-SMC on days 0, 7 or 14. Red arrows show ischemic myocytes with plump and centralized nuclei. Hematoxylin and eosin stain; 40× magnification. (**B**) Bar graph of muscle fiber number (two-way ANOVA, *p* < 0.0001; *n* = 5, **** *p* < 0.0001 (post-hoc)). (**C**) Bar graph of muscle fiber area (two-way ANOVA, *p* = 0.0001; *n* = 5, * *p* < 0.05, ** *p* < 0.01, *** *p* < 0.001, **** *p* < 0.0001 (post-hoc)).

Histological findings correlated with functional outcomes (Figure 4). hiPSC-SMC- and ADSC-treated mice showed fewer muscle fibers per high-power field compared with control mice at day 7 (hiPSC-SMC, 70.6 ± 1.8, *p* < 0.0001; ADSC, 74.0 ± 2.1, *p* < 0.0001; Figure 4A,B) and day 14 (hiPSC-SMC, 66.2 ± 2.0, *p* < 0.0001; ADSC, 67.6 ± 2.1, *p* < 0.0001; Figure 4A,B). In addition, hiPSC-SMC- and ADSC-treated mice showed increased gastrocnemius muscle fiber area compared with control mice at day 7 (hiPSC-SMC, 989 ± 51 μm^2^, *p* < 0.01; ADSC, 953 ± 32 μm^2^, *p* < 0.05; Figure 4A,B) and day 14 (hiPSC-SMC, 1180 ± 16 μm^2^, *p* < 0.001; ADSC, 1119 ± 67 μm^2^, *p* < 0.001; Figure 4A,C). Gastrocnemius muscle from the ischemic limb of hiPSC-SMC- and ADSC-treated mice appeared healthy with polygonal myocytes and peripheral nuclei; conversely, gastrocnemius muscle from the ischemic limb of control mice showed ischemic myocytes that were contracted with loss of polygonal shape and centralized nuclei (Figure 4A).

### 3.3. hiPSC-SMC Stimulate Angiogenesis

**Figure 5:**
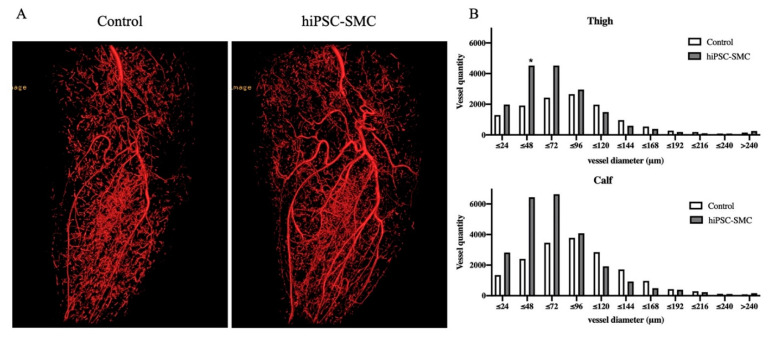
hiPSC-SMC increase angiogenesis. (**A**) Representative microCT images of a mouse leg treated with control (left panel) or hiPSC-SMC (right panel), day 14. (**B**) Representative data showing vessel quantity in the thigh (upper graph) or calf (lower graph), day 14 (Student’s *t*-test, * *p* < 0.05; *n* = 2).

**Figure 6:**
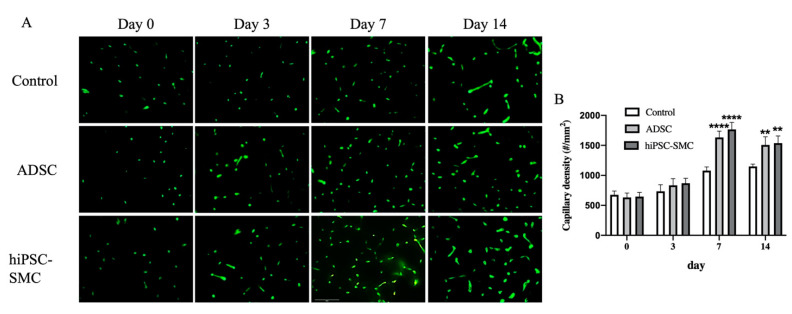
hiPSC-SMC increase capillary density in gastrocnemius muscle. (**A**) Representative immunofluorescence of isolectin B4 in gastrocnemius muscle in mice treated with control, ADSC or hiPSC-SMC on days 0, 3, 7 or 14. (**B**) Bar graph of capillary density (two-way ANOVA, *p* < 0.0001; *n* = 5, ** *p* < 0.01, **** *p* < 0.0001 (post-hoc)).

To determine whether the improved ischemia in hiPSC-SMC-treated mice reflects increased angiogenesis, we directly examined angiogenesis in ischemic limbs using microCT angiography (Figure 5A). There were more arterioles less than 96 μm in diameter in the hiPSC-SMC-treated limbs (Figure 5B). To confirm the microCT data, we quantified capillary density using immunofluorescence (Figure 6). hiPSC-SMC-treated mice showed increased gastrocnemius capillary density compared with control mice at day 7 (hiPSC-SMC, 1766 ± 52, *p* < 0.0001; ADSC, 1633 ± 47, *p* < 0.0001) and day 14 (hiPSC-SMC, 1538 ± 53, *p* < 0.01; ADSC, 1508 ± 60, *p* < 0.01). The capillaries were distributed between muscle bundles. These data show increased angiogenesis in hiPSC-SMC- and ADSC-treated mice compared with control mice, consistent with the data showing increased limb perfusion.

### 3.4. hiPSC-SMC Treatment is Associated with Fewer Macrophages

**Figure 7:**
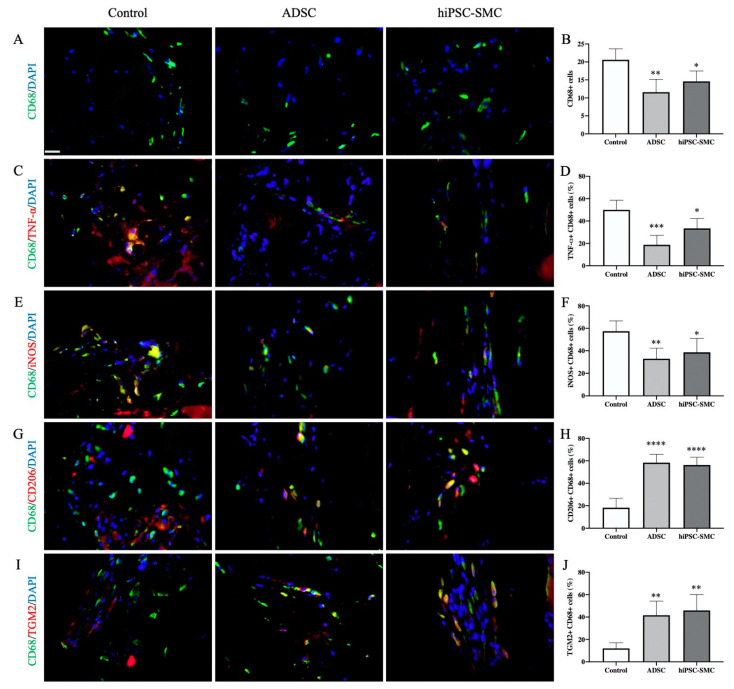
hiPSC-SMC decrease M1-type macrophages and increase M2-type macrophages. (**A**) Representative immunofluorescence of gastrocnemius muscle (day 3) showing CD68-positive cells (green) in control (left), ADSC (middle) and hiPSC-SMC (right) groups; DAPI, blue; scale bar, 20 μm. (**B**) Bar graph showing the number of CD68-positive cells per high power field; * *p* < 0.05, ** *p* < 0.01 (ANOVA); *n* = 5. (**C**) Representative immunofluorescence of gastrocnemius muscle (day 3) showing immunoreactivity with CD68 (green) and TNF-α (red); DAPI, blue. (**D**) Bar graph showing percentage of TNF-α/CD68 dual-positive cells; * *p* < 0.05, *** *p* < 0.001 (ANOVA); *n* = 5. (**E**) Representative immunofluorescence of gastrocnemius muscle (day 3) showing immunoreactivity with CD68 (green) and iNOS (red); DAPI, blue. (**F**) Bar graph showing percentage of iNOS/CD68 dual-positive cells; * *p* < 0.05, ** *p* < 0.01 (ANOVA); *n* = 5. (**G**) Representative immunofluorescence of gastrocnemius muscle (day 3) showing immunoreactivity with CD68 (green) and CD206 (red); DAPI, blue. (**H**) Bar graph showing percentage of CD68/CD206 dual-positive cells; **** *p* < 0.0001 (ANOVA); *n* = 5. (**I**) Representative immunofluorescence of gastrocnemius muscle (day 3) showing immunoreactivity with CD68 (green) and TGM2 (red); DAPI, blue. (**J**) Bar graph showing percentage of CD68/TGM2 dual-positive cells; ** *p* < 0.01 (ANOVA); *n* = 5.

Next, we determined the numbers of macrophages as well as the macrophage phenotype that was associated with each treatment group. Both hiPSC-SMC and ADSC treatments were associated with decreased numbers of CD68-positive cells compared with the control treatment (hiPSC-SMC, 14.6 ± 1.3, *p* < 0.05; ADSC, 11.6 ± 1.6, *p* < 0.01; day 3; Figure 7A,B), consistent with fewer macrophages. There were fewer numbers of CD68/TNFα dual-positive cells (hiPSC-SMC, 33.4 ± 3.9%, *p* < 0.05; ADSC, 18.8 ± 3.8%, *p* < 0.001; Figure 7C,D) as well as CD68/iNOS dual-positive cells (hiPSC-SMC, 38.7 ± 5.5%, *p* < 0.05; ADSC, 33.0 ± 4.2%, *p* < 0.01; Figure 7E,F) in ischemic limbs treated with hiPSC-SMC or ADSC compared with control treatment, suggesting fewer M1-type macrophages. However, there were increased numbers of CD68/CD206 dual-positive cells (hiPSC-SMC, 56.3 ± 3.1%, *p* < 0.0001; ADSC, 58.4 ± 3.3%, *p* < 0.0001; Figure 7G,H) as well as CD68/TGM2 dual-positive cells (hiPSC-SMC, 45.9 ± 6.3%, *p* < 0.01; ADSC, 41.6 ± 5.6%, *p* < 0.01; Figure 7I,J) in limbs treated with hiPSC-SMC or ADSC compared with control treatment, consistent with increased numbers of M2-type macrophages. M2-type macrophages were mainly distributed between muscle bundles and in intermuscular spaces. These data suggest that treatment of ischemic limbs with hiPSC-SMC is associated with fewer macrophages, decreased numbers of M1-type macrophages, and increased numbers of M2-type macrophages.

### 3.5. hiPSC-SMC Increase Angiogenesis by Promoting VEGF-A Secretion

**Figure 8:**
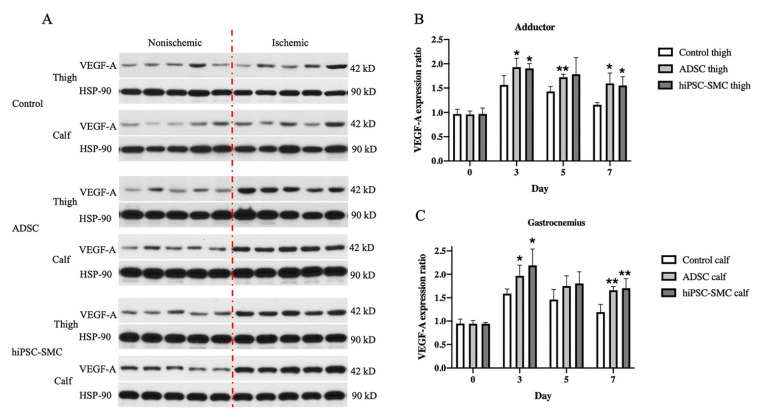
hiPSC-SMC increase VEGF-A expression. (**A**) Western blot of vascular endothelial growth factor (VEGF)-A at day 3. (**B**) Bar graph shows the VEGF-A expression ratio (ischemic limb (right) to the nonischemic hind limb (left)) in the adductor muscle of mice treated with control, ADSC or hiPSC-SMC, at days 0, 3, 5 and 7; two-way ANOVA, *p* < 0.01; *n* = 5, * *p* < 0.05, ** *p* < 0.01 (post-hoc). (**C**) Bar graph shows the VEGF-A expression ratio (ischemic limb (right) to the nonischemic hind limb (left)) in the gastrocnemius muscle of mice treated with control, ADSC or hiPSC-SMC on days 0, 3, 5 and 7; two-way ANOVA, *p* < 0.0001; *n* = 5, * *p* < 0.05, ** *p* < 0.01 (post-hoc).

**Figure A3:**
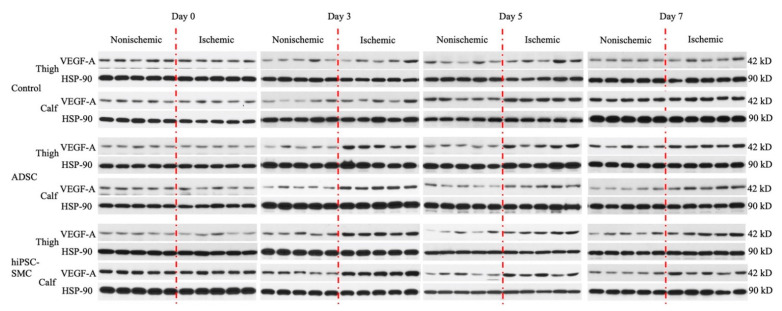
VEGF-A expression of Control, ADSC, and hiPSC-SMC groups at day 0, 3, 5 and 7.

Next, we determined whether expression of VEGF-A was a potential mechanism of increased angiogenesis in hiPSC-SMC-treated mice (Figure 5 and Figure 6). VEGF-A expression was significantly elevated in both the adductor muscle and the gastrocnemius muscle of ischemic limbs in both hiPSC-SMC- and ADSC-treated mice compared with control treatment (day 3, *p* < 0.05; Figure 8A,C). VEGF-A expression was maximal at day 3 and then gradually decreased in all three groups (Figure 8 and Figure A3).

**Figure 9:**
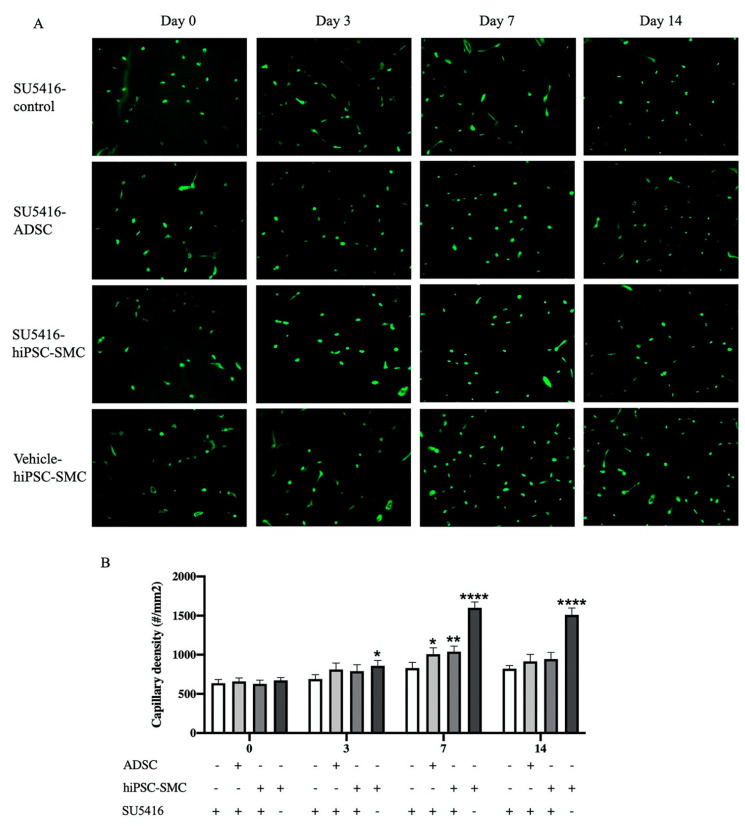
Capillary density in gastrocnemius muscle of control (SU5416-control), ADSC (SU5416-ADSC) and hiPSC-SMC (SU5416-hiPSC-SMC) groups with administration of VEGFR2 inhibitor and hiPSC-SMC group with vehicle only (vehicle-hiPSC-SMC) at day 0, 3, 7 and 14. (**A**), Immunofluorescent isolectin B4 staining of ischemic gastrocnemius. (**B**), Quantification of capillary density in the ischemic limbs. (two-way ANOVA, *p* < 0.0001; *n* = 5, * *p* < 0.05, ** *p* < 0.01, **** *p* < 0.0001 (post-hoc)). -, absence; +, presence.

To determine whether immunoreactive VEGF-A was functional and thus could be a mechanism of the angiogenic potential of hiPSC-SMC, SU5416, a potent and selective inhibitor of the VEGF receptor 2 tyrosine kinase, or vehicle control were injected daily intraperitoneally. The positive control group mice treated with both hiPSC-SMC and vehicle control showed increased angiogenesis, as expected; compared with this group, there was lower capillary density in hiPSC-SMC-treated mice also administered with SU5416 (day 14, *p* < 0.0001; Figure 9). ADSC-treated mice similarly administered with SU5416 also showed diminished capillary density (*p* < 0.05; Figure 9). These data suggest that increased VEGF-A expression is a mechanism by which hiPSC-SMC improve angiogenesis.

## 4. Discussion

This study showed that hiPSC-SMC improve hindlimb ischemia and promote angiogenesis. hiPSC-SMC treatment was associated with fewer macrophages, fewer M1-type macrophages, and a greater percentage of M2-type macrophages. Increased VEGF-A expression appeared to be responsible for the increased angiogenesis in hiPSC-SMC-treated mice. These data show that hiPSC-SMC attenuate hindlimb ischemia in a mouse model, suggesting their translational potential to human patients.

Our primary finding showed that hiPSC-SMC improve hindlimb ischemia and stimulate angiogenesis. Patients with no-option CLTI represent a major therapeutic challenge and are traditionally associated with a poor prognosis; administration of cell populations capable of angiogenesis may result in improved perfusion and represent a novel strategy to improve outcomes. In a mouse model of hindlimb ischemia, endothelial progenitor cells can form neovessels [33]. Implantation of autologous bone-marrow-derived mononuclear cells in the gastrocnemius of an ischemic leg reduced rest pain and increased transcutaneous oxygen pressure in patients with CLTI [34]. A randomized controlled trial showed that intramuscular injection of autologous bone-marrow-derived MSC improved symptoms, accelerated ulcer healing, and increased limb perfusion in diabetic patients with CLTI [9]. iPSC represent a novel and renewable cell source without considerable ethical issues. Thus far, hiPSC-EC and hiPSC-derived MSC have improved blood flow and reduced muscle fibrosis and tissue loss in murine models [16,17,35,36]. Our data showing that hiPSC-SMC stimulate angiogenesis in a hindlimb ischemia model agree with recent data reported by Park et al. [37], who use a similar animal model. The femoral artery transection model and the femoral/saphenous artery excision model are the two most commonly used murine models of unilateral hind limb ischemia. The former produces only a mild-to-moderate amount of ischemia that induces mainly arteriogenesis in the thigh, with minimal calf angiogenesis. The latter femoral/saphenous artery excision model produces more severe ischemia that induces both thigh arteriogenesis as well as calf angiogenesis [24]. We used the more severe femoral/saphenous artery excision model to facilitate the evaluation of translational potential. However, the applicability of acute limb ischemia models to human patients with chronic limb-threatening ischemia may be limited, and additional experiments showing the utility of hiPSC-SMC in better models are warranted.

Our data also showed that hiPSC-SMC treatment was associated with fewer macrophages and a reduced percentage of M1-type macrophages but an increased percentage of M2-type macrophages. M1-type macrophages are generally considered to be proinflammatory and cytotoxic whereas M2-type macrophages are typically immunosuppressive and proangiogenic. Polarization of macrophages to the M2-type phenotype promotes angiogenesis and is a potential therapeutic approach to ischemic diseases [38,39]. Macrophages support angiogenesis, not only by secreting proangiogenic factors and matrix-remodeling proteases but also by physical cooperation with sprouting vasculature [40]. MSC that increased the infiltration of M2-type macrophages into ischemic legs facilitated fast functional recovery and promoted angiogenesis in mouse models [41,42]. Our findings of decreased M1-type and increased M2-type macrophages with both hiPSC-SMC and ADSC suggest that hiPSC-SMC and ADSC treatment may shift the M1–M2 phenotypic switch at an earlier time point, compared with control treatment. It is also possible that the increased M2-type macrophages may be a useful surrogate marker for angiogenesis that occurs during therapy for hindlimb ischemia.

Our data also showed that increased VEGF-A expression may be a mechanism by which hiPSC-SMC improve angiogenesis. Angiogenic factors such as VEGF, fibroblast growth factor (FGF), and hepatocyte growth factor (HGF) have been used to treat patients with claudication or CLTI in clinical studies [43,44,45,46]. However, there is a lack of consistent and demonstrable benefit with specific factors. Stem cells that have the ability to self-renew and differentiate into specific cells and also stimulate paracrine effects by releasing multiple pro-angiogenic factors and cytokines have emerged as an alternative treatment option. One study showed that iPSC-EC secreted significantly more major angiogenic factors, including VEGF, HGF and EGF, during hypoxia, compared with normoxia in vitro, and significantly attenuated severe hind-limb ischemia in mice via enhancement of neovascularization [47]. Our findings of elevated expression of VEGF-A in ischemic muscle and diminished capillary density after administration of SU5416 suggest that increased VEGF-A expression appears to be a mechanism by which hiPSC-SMC improve angiogenesis. Nevertheless, other factors, such as hiPSC-SMC-derived exosomes, may be another mechanism of action [37].

Although our study shows that hiPSC-SMC improve hindlimb ischemia in a murine model, there are several limitations. A possible obstacle to the clinical translation of iPSC therapy into human patients is the potential risk of oncogenic transformation of undifferentiated cells [48]. We used hiPSC-SMC rather than hiPSC-MSC, since we can generate large numbers of hiPSC-SMC via a highly effective approach that generates SMC with high purity, which should minimize or eradicate such a potential risk [23], and we have also used them for other translational applications [18,23]. However, other hiPSC-derived cell types, such as hiPSC-MSC, might be easier to generate for translational applications and need additional study. iPSC and their differentiated derivatives may have the potential of carrying genomic abnormalities and undergoing cellular transformation [49]. Careful genotyping during cell culture should be able to ensure normal chromosomes in iPSC and iPSC-derived progenies. In addition, this study only examined male animals and measured benefit within only 14 days; further studies on female animals and with a longer follow-up are necessary to validate these findings. Lastly, although our study showed that hiPSC-SMC promote VEGF-mediated angiogenesis that improves hindlimb ischemia, other mechanisms for improved hindlimb ischemia remain to be determined. Despite these limitations, our study has identified hiPSC-SMC as a potential cellular source to improve hindlimb ischemia.

## 5. Conclusions

hiPSC-SMC promote VEGF-mediated angiogenesis, leading to improved hindlimb ischemia. Stem cell therapy using iPSC-derived cells is a novel and potentially translatable therapy for limb-threatening ischemia.

## Figures and Tables

**Table 1:** Functional scoring.

Score	Description
Tarlov score	
0	No movement
1	Barely perceptible movement, non–weight-bearing
2	Frequent movement, non–weight-bearing
3	Supports weight, partial weight-bearing
4	Walks with mild deficit
5	Normal but slow walking
6	Full and fast walking
Faber ischemia score	
0	Normal
1–5	Cyanosis or loss of nail(s)
6–10	Partial or complete atrophy of digit(s)
11–12	Partial atrophy or gangrene of forefoot

## Data Availability

All data presented in this study is present within the manuscript.

## Appendix A

**Table A1:** List of Primers for qRT-PCR.

Gene	Primer Sequence
GAPDH-F	TGTTGCCATCAATGACCCCTT
GAPDH-R	CTCCACGACGTACTCAGCG
α-SMA-F	CTGGGACGACATGGAAAA
α-SMA-R	ACATGGCTGGGACATTGA
CNN1-F	AGCATGGCGAAGACGAAAGGAA
CNN1-R	CCCATCTGCAGGCTGACATTGA
MYH11-F	AGAGACAGCTTCACGAGTATGAG
MYH11-R	CTTCCAGCTCTCTTTGAAAGTC
OCT-4-F	GCAGCTCGGAAGGCAGAT
OCT-4-R	TGGATTTTAAAAGGCAGAAGACTTG

**Table A2:** List of antibodies for immunostaining.

Antigen	Catalog #	Company	Dilution Used
MYH11	ab53219	Abcam	1:100
α-SMA	A5228	Sigma	1:100
CNN1	C2687	Sigma	1:100
OCT4	ab18976	Abcam	1:100
isolectin	DL-1208	Vector	1:200
CD68	MCA1957	BioRad	1:200
iNOS	ab15323	Abcam	1:200
CD206	ab64693	Abcam	1:200
TGM2	CST3557	Cell Signaling	1:200
goat anti-rabbit Alexa-Fluor 568	A-11011	Invitrogen	1:200–1:500
goat anti-mouse Alexa-Fluor-568	A-11004	Invitrogen	1:200–1:500
goat anti-rat Alexa-Fluor-568	A-11077	Invitrogen	1:200–1:500
goat anti-mouse Alexa-Fluor-488	A-11001	Invitrogen	1:200–1:500
goat anti-rat Alexa-Fluor-488	A-11006	Invitrogen	1:200–1:500
